# Real-time streaming tomographic reconstruction with on-demand data capturing and 3D zooming to regions of interest

**DOI:** 10.1107/S1600577522003095

**Published:** 2022-04-20

**Authors:** Viktor Nikitin, Aniket Tekawade, Anton Duchkov, Pavel Shevchenko, Francesco De Carlo

**Affiliations:** a Advanced Photon Source, Argonne National Laboratory, Lemont, IL 60439, USA; b Data Science and Learning Division, Argonne National Laboratory, Lemont, IL 60439, USA; c Institute of Petroleum Geology and Geophysics SB RAS, 630090 Novosibirsk, Russia; d Novosibirsk State University, 630090 Novosibirsk, Russia

**Keywords:** micro-tomography, streaming imaging, real-time reconstruction, 3D zooming, multi-scale tomography

## Abstract

Complex dynamic tomographic experiments at brilliant X-ray light sources require real-time feedback on the sample changes with respect to environmental conditions, selecting representative regions of interest for high-resolution scanning, and on-demand data saving mechanisms for storing only relevant projections acquired by fast area detectors and reducing data volumes. Here the implementation details of a 3D real-time imaging monitoring instrument, with zooming to a volume of interest with easy-to-use visualization via *ImageJ*, a tool familiar to most beamline users, is presented. The instrument relies on optimized data flow between the detector and processing machines and is implemented on commodity computers. The instrument has been developed at beamline 2-BM of the Advanced Photon Source, where the automatic lens changing mechanism for zooming is implemented with an Optique Peter microscope. Performance tests demonstrate the ability to process more than 3 GB of projection data per second and generate real-time 3D zooming with different magnification. These new capabilities are essential for new APS Upgrade instruments such as the projection microscope under development at beamline 32-ID. The efficacy of the proposed instrument was demonstrated during an *in situ* tomographic experiment on ice and gas hydrate formation in porous samples.

## Abstract 2

A new instrument for real-time reconstruction of three arbitrary slices of raw tomographic projection data streaming from a 2D detector is presented. The instrument allows for quick 3D zooming to regions of interest defined by the intersection of the reconstructed slices, data capturing to HDF5 files whenever required, and changing of all data collection parameters during the streaming.

## 1. Introduction

The development of high-speed time-resolved tomographic microscopy is of great interest for various three-dimensional (3D) *in situ* studies including material science (Maire *et al.*, 2016 ▸; Zhai *et al.*, 2019 ▸), geology (Butler *et al.*, 2020 ▸; Nikitin *et al.*, 2020 ▸), and energy sources (Finegan *et al.*, 2015 ▸; Liu *et al.*, 2019 ▸). Brilliant synchrotron light sources are able to perform continuous tomographic data acquisition at a rate of more than 7.7 GB s^−1^ (Mokso *et al.*, 2017 ▸; García-Moreno *et al.*, 2021 ▸), generating terabytes of data in a very short time, opening the possibility of studying very fast processes at unprecedented high temporal resolution. Most of the current high-speed tomographic instruments capture events in a preset region of the sample and monitor the sample evolution by only looking at projection data. In most cases, this semi-blind conventional approach leads to missing the dynamic phenomenon as the place and time of its origination may not be known in advance.

One of the main difficulties in studying fast processes is to select a representative region of interest for scanning, *i.e.* the region where the dynamic process starts occurring and evolves in time. In most cases the dynamic phenomenon is not captured because it occurs in a location not under observation, is faster than expected or requires a different spatial or temporal resolution than the one the instrument is configured at. Another difficulty in *in situ* studies of dynamically changing samples is the determination of the optimal environmental control system conditions. Without real-time 3D imaging feedback, it is nearly impossible to set optimal environmental conditions, such as cooling temperature, pressure or loading forces, especially when the X-ray beam itself affects the sample state.

There are many studies that would greatly benefit from fast 3D imaging optimized by using real-time image reconstruction for feedback and control.

In material engineering and geomechanics it is important to understand mechanisms of failure origination. But these processes are very challenging for 3D imaging because a crack may start in different parts of the sample. A recent study demonstrates that it is possible to follow a crack propagation in 3D at a 20 Hz acquisition rate and a sample-rotation rate of 10 rev min^−1^ (Maire *et al.*, 2016 ▸). However, the chance of successful imaging of the crack is only 5%. Marti *et al.* (2021 ▸) studied hydrofracturing in gypsum and reported a possibility to perform 3D imaging at a rate of 2 s per full scan. But in most cases the fast process of rock failure is still studied using comparatively slow imaging. Processes of rupture nucleation, evolution of damage and strain localization in tri-axially compressed porous limestone were imaged at a rate of only 3 min for one full scan (Renard *et al.*, 2017 ▸; Huang *et al.*, 2019 ▸). Studying mechanisms of rock failure is important to better understand hydraulic fracturing but we did not find studies utilizing real-time 3D imaging as a routine tool. Voltolini & Ajo-Franklin (2020*a* ▸) show a fracture sealing mechanism in clay, revealed by *in situ* 3D imaging at a rate of ∼14 min for one full scan. An important process of evolution of propped fractures in shale was imaged only at a rate of ∼10 min for one full scan (Voltolini & Ajo-Franklin, 2020*b* ▸); fracture development caused by the freeze–thaw cycling of porous samples was imaged at a rate of 1.3 min for one scan (De Kock *et al.*, 2015 ▸).

An important topic in geosciences is to study fast non-equilibrium pore-scale processes including wetting, dilution, mixing, and reaction phenomena, without sacrificing significant spatial resolution. For example, fast pore-scale fluid dynamics – an incremental capillary-water movement known as the Haines jumps – was observed in *in situ* experiments with 3D imaging every 16 s (Berg *et al.*, 2013 ▸; Singh *et al.*, 2018 ▸), and at a 20 Hz rate (Dobson *et al.*, 2016 ▸). Such results form the basis for developing and validating multi-scale flow models through heterogeneous pore networks. Note that fast imaging in the latter case is not continuous but interrupted by data transfer on the disk. An important process of super-critical CO_2_ flow in porous rocks for carbon sequestration and storage was imaged only at a rate of 2.5 min for one scan (Voltolini *et al.*, 2017 ▸). Nikitin *et al.* (2020 ▸) used dynamic *in situ* imaging at a rate of 70 s per full scan to study the process of methane hydrate formation in porous samples. They mentioned that this scanning rate was not sufficient for studying the faster hydrate dissociation processes and reported fluid movement artifacts in the images. Fast time-resolved tomographic experiments to study bubble growth in basaltic foam were conducted by Baker *et al.* (2012 ▸) where the authors reported that a temporal resolution issue caused by rapid sample movements caused the fast dynamics of earliest bubble growth. They were acquiring data with a fast camera at a rate of 1 s per 180° interval, with 18 s total time for a measurement. Because of sample motion they reported only the measurements commencing 10–11 s following the earliest start of the bubble growth. In the following work (Pleše *et al.*, 2018 ▸), the authors performed scanning with 0.5 s per 180° interval and were able to acquire 100 tomographic datasets for each measurement. In order to capture the initiation of the bubbles growth, the authors monitored radiographs (2D projection images) of one section of each sample and started tomographic data acquisition according to the approximate temperature inside the sample.

A conventional approach for data acquisition in tomographic experiments is based on real-time visualization of 2D projections streamed from the detector. These projections are typically used to align the sample on the rotation stage and adjust the detector exposure time. Further tomographic scanning in fly scan mode (Wang *et al.*, 2019 ▸) involves saving a series of projections while the sample is continuously rotated over a 180° interval. After scanning, the acquired data are transferred from the detector computer to a processing and visualization workstation where the reconstruction procedure and the 3D rendering are performed. Data acquisition and reconstruction becomes time-consuming especially in the case of dynamic tomography experiments. At the micro-tomography beamline 2-BM of the Advanced Photon Source the typical acquisition time for geological samples is about 1–3 min, depending on the sample thickness and absorption; nano-tomography measurements at sector 32-ID (De Andrade *et al.*, 2021 ▸), in turn, take 15–30 min (Nikitin *et al.*, 2021 ▸). At both beamlines, the reconstruction time of a full 3D volume using *tomoPy* (Gürsoy *et al.*, 2014*a* ▸) multi-threaded CPU-based functions is about 15 min.

The acceleration of tomographic reconstruction with modern multi-CPU and multi-GPU systems has always been of great interest in the imaging community. Parallel implementations on multi-core CPU clusters (Bicer *et al.*, 2017 ▸; Marone *et al.*, 2017 ▸) are based on splitting data over slices and processing them independently on different nodes. Similarly, GPU implementations with CUDA technology (van Aarle *et al.*, 2015 ▸; Andersson *et al.*, 2016 ▸) allows for reconstructing full 3D volumes in less than a minute. While these approaches demonstrate significant reduction in computational times, reconstruction of full 3D volumes remains challenging in the case of real-time tomographic reconstruction. In most cases though, beamline users primarily look at recovered 3D volumes slice by slice while the experiment is ongoing. More detailed analysis involving, for instance, segmentation and quantification is typically done after the experiment. The computational cost to reconstruct a set of arbitrary slices through a sample is negligible compared with a full volume reconstruction. In many cases, a set of arbitrary slices are sufficient to implement real-time instrument feedback and to enable users to make decisions about data quality and dynamic event capturing (Buurlage *et al.*, 2018 ▸, 2019 ▸).

Current frameworks for controlling detectors allow for pipelining complex data flows including real-time projection processing on different devices. Perhaps the most advanced interface is provided by EPICS *areaDetector* (Rivers *et al.*, 2010 ▸; Rivers, 2017 ▸), where a set of plugins running in their own threads allows for real-time processing of detector raw data. The list of plugins (Rivers, 2022*a* ▸) includes statistics calculations, image processing, region of interest extraction, file saving, and exporting images via EPICS Channel Access or pvAccess (Veseli, 2015 ▸) for display in clients like the *ImageJ* package (Schneider *et al.*, 2012 ▸), a commonly used software in the imaging community, by making use of the EPICS NTNDA Viewer (Rivers, 2022*b* ▸) plugin, or further processing.

In this paper we propose a real-time 3D imaging monitoring instrument able to: (1) optimize alignment and data collection parameters including exposure time and angular step size while streaming tomographic projections are collected in fly scan mode; (2) trigger data save on-demand at any time while continuing the streaming monitoring; (3) add to the saved data an arbitrary set of projections streamed before the data save trigger event; (4) perform real-time tomographic reconstruction of three arbitrary slices across the sample; (5) perform a three-lens zoom-in/zoom-out to a region of interest centered on the intersection point of the three arbitrary slices; and (6) swap detector when different frame rate or pixel size are required. The proposed instrument has been implemented at beamline 2-BM of the Advanced Photon Source, where the automatic lens changing mechanism for zooming was realized by an Optique Peter microscope system (Optique-Peter, 2022 ▸). We demonstrate real-time streaming reconstruction and *ImageJ* visualization of three arbitrary slices through the sample volume, as well as optimal data flow management for streaming reconstruction and projection capturing to HDF5 files with EPICS *areaDetector*. To prove the efficacy of the proposed streaming functionality in providing effective real-time feedback, we include detailed performance tests for reconstruction using commodity computers, and demonstrate its use in an *in situ* tomographic experiment of ice and gas hydrate formation in porous media.

## 2. Streaming data acquisition model

We define data streaming as a transmission or broadcast over the network of images as structured objects that include metadata sufficient to correctly handle transmission errors. For example, in the case of tomographic projection images, these metadata include the angle at which that projection was collected. The proposed streaming data acquisition model consists of the following parts:

(1) Broadcasting projections over the network in streaming mode with on-demand data capturing to disk.

(2) Changing acquisition parameters, including exposure time and angular step, while in streaming mode.

(3) Real-time streaming reconstruction and visualization of three arbitrary slices through the sample.

(4) 3D zooming to regions of interest inside the sample, while in streaming mode.

In the proposed model we assume fly scan data collection, *i.e.* the studied sample is positioned on a continuously spinning rotary stage and that its encoder pulses are used to trigger the detector whenever each particular projection angle should be acquired. The specific hardware used to demonstrate this model is listed in Section 3.

### 2.1. Broadcasting projections over the network in streaming mode with on-demand data capturing to disk

Projection data broadcasting and capturing relies on using the PVA1 pvAccess plugin (NDPluginPva, 2022 ▸) of *areaDetector* (*AD*), an application for controlling area (2D) detectors, including CCDs, pixel array detectors, and online imaging plates (Rivers, 2017 ▸). We utilize the PVA1 plugin to form structured objects containing the projection data and transport them over the network. Besides the 2D raw image array, the structured object includes information about data size, binning, frame type, and a unique ID for each projection. Frame type is assigned the values of ‘Projection’, ‘DarkField’, and ‘FlatField’, and is used to distinguish raw projection data, dark-field data (when the beam is off), or flat-field data (when the beam is on but the object is out of the field of view) to further apply dark-flat-field correction in the reconstruction procedure. The reconstruction engine directly converts the unique ID of each projection to the rotation angle at which the projection is acquired. If, during streaming, a projection is missed, due to slow network connection, transmission errors, *etc*., the corresponding angle will be skipped as well and the error be handled with negligible or no effect on the real-time reconstruction quality. The *AD* region of interest (ROI) plugin ROI1 is used for data cropping and binning. The capturing of data from the detector stream to disk is handled by the *AD* HDF plugin which, besides raw data, also saves all metadata associated with the tomographic scan using the data-exchange (De Carlo, 2022*a* ▸) file format. To implement the data-exchange schema, the *AD* HDF plugin is configured using *ad hoc* xml files (De Carlo, 2022*b* ▸). In addition, we utilize the *AD* circular buffer (NDPluginCircularBuff, 2022 ▸) plugin CB1 to store a set of projections back in time. In the case of dynamic tomography, the circular buffer allows users to capture the beginning of the studied dynamic process and monitor the whole process evolution.

Plugins run in their own threads, allowing parallel processing on multi-core machines. All *AD* plugins can be structured as a set of independent pipelines. In the proposed streaming implementation we have two pipelines reading data from the same detector channel RAW DATA:[Chem scheme1] 
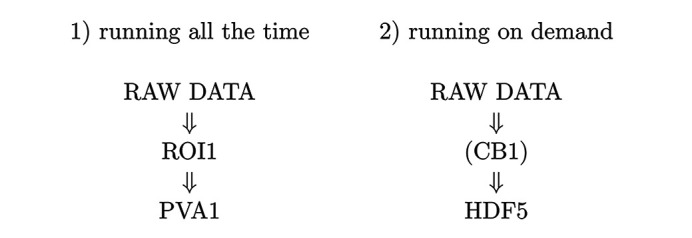


The first pipeline prepares data for broadcasting and streaming reconstruction. In case of a slow network connection or limited computing resources, one can apply binning or crop data to smaller sizes by adjusting parameters in the ROI1 plugin. The second pipeline is associated with on-demand data capturing to an HDF5 file located on the machine controlling the detector. As opposed to the first pipeline, no data binning or cropping is applied in this case.

Dark and flat fields are acquired on-demand while the sample is continuously rotated. Dark and flat data are saved in temporary HDF5 files named ‘dark_fields.h5’ and ‘flat_fields.h5’, and copied to any future HDF5 file with projection data whenever projection capturing is executed. Each resulting HDF5 file containing dark, flat, and projection data is structured as a standard Data Exchange file (De Carlo *et al.*, 2014 ▸) and can be read using the read_dx() function from the *DXchange* toolbox (De Carlo, 2022*a* ▸). These files can be reconstructed offline using *tomoPy* (Gürsoy *et al.*, 2014*b* ▸) via its command-line interface *tomopyCLI* (De Carlo, 2019 ▸), or using a GPU-based reconstruction with *tomocupyCLI* (Nikitin, 2022 ▸).

Recollecting the dark and flat fields while in streaming mode will update the ‘dark_fields.h5’ and ‘flat_fields.h5’ files. Each set of dark and flat fields is averaged, down-sampled with respect to binning defined in the ROI1 plugin, and broadcasted over the network via pvAccess variables. In this way, any connected reconstruction engine has access to the most recently collected dark and flat fields.

The circular buffer stores projection data representing the state of the sample earlier in time, before the on-demand projection capturing starts. Since the *AD* HDF5 file plugin does not allow appending projections at the beginning of the projection array in the file, one has to implement additional data merging procedures involving large data transfers inside memory. To avoid this, we decided to store the circular buffer in a separate HDF5 file and apply an offline projection array merging whenever streaming is done. Alternatively, one can consider using virtual HDF5 datasets that allow several real datasets to be mapped together into a single and sliceable dataset via an interface layer.

We implemented the projection broadcast and on-demand data capturing to disk described above in the *tomoScanStream* Python class which is a part of *tomoScan* (Rivers, 2022*c* ▸).

### 2.2. Changing acquisition parameters, including exposure time and angular step, while in streaming mode

An additional important feature of the proposed streaming model is the possibility to change data acquisition parameters, such as exposure time and angular step, without stopping the streaming process. For instance, exposure time needs to be adjusted when changing energy or magnification setups using thicker (thinner) or more (less) efficient scintillators required to achieve a desired spatial resolution or data collection speed. Moreover, fast real-time streaming reconstruction of sample slices with very short exposure time and sparse angles is often required for quick ROI search and for monitoring the sample dynamics, whereas data capturing to an HDF5 file, in most cases, should be done with optimal acquisition parameters. Exposure time and angular step change involves accelerating or decelerating the rotation stage while the stage is moving. In our measurements we used an Aerotech stage model ABRS-150MP-M-AS with the Aerotech Ensemble Controller model HLE10-40-A-MXH operated by using commands from Aerobasic programming language. The controller allows for changing the rotation speed without stopping the stage only when the rotation is done in the ‘Jog’ motion mode. When the stage is rotating, its encoder sends trigger pulses to the detector whenever each subsequent projection needs to be acquired. To change the rotation speed without losing track of the unique image ID and its matching angular position, while the rotary stage is moving, we implemented a sequence which (1) temporarily terminates the trigger pulses (locking the unique image ID), (2) changes the rotation speed of the ‘Jog’ motion, (3) arms the recording of the stage encoder value using the Aerobasic commands in the DATAACQ group, then (4) resumes sending pulses.

The first recorded encoder pulse allows for recovering the new rotary stage angular position at which the first projection is acquired after changing the rotation speed. This image unique ID is known (equal to the locked unique ID + 1) and allows for re-synchronizing images with the new angular positions. With the initial angle and the new angular step we form a new array of angles and broadcast it as a PV variable used by the streaming reconstruction. The reconstruction engine is reinitialized whenever a new set of angles is generated. The whole procedure for rotation change with corresponding Aerobasic code parts can be summarized as follows:

(1) Initiate ‘Jog’ rotation with velocity based on exposure time and angular step.

(2) Wait for rotation stage acceleration time.

(3) Program PSO:


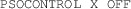
 – turns off pulses.


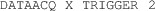
 – specifies which signal is monitored to collect data.


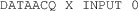
 – specifies the data element collected when a trigger occurs.


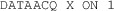
 – enables data collection.


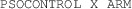
 – turns on pulses.

(4) Wait when one pulse is acquired.

(5) Read encoder value:


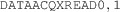
 – read one encoder value to an integer register.


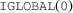
 – read value from the register.

(6) Turn off encoder values acquisition:


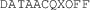
 – disable data collection.

(7) Convert encoder value to the angle, match the angle with projection unique ID.

(8) Create a new array of angles and broadcast it in a PV variable.

### 2.3. Real-time streaming reconstruction and visualization of three arbitrary slices through the sample

Each reconstruction engine in the streaming model is associated with a computer equipped with a GPU. It continuously performs the following operations: (1) reading structured projection data from the network via the PVA1 plugin of *AD*, (2) fast reconstruction of three arbitrary slices on GPU, and (3) broadcasting the result over the network as a pvAccess variable. To implement the concurrent execution of these operations we utilize multi-threading implemented with regular Python threads.

The first thread reads the structured projection data (containing unique projection ID) from the network and adds it to a synchronized buffer with a given total number of items. Typically the buffer size corresponds to the number of projections in a 180° interval yielding a full tomographic reconstruction. However, to improve reconstruction quality of noisy data in some cases it makes sense to consider several 180° intervals for processing. New incoming projections replace the buffer elements corresponding to the same angles at which these new projections were acquired. The new buffer elements are consumed by the reconstruction engine to update three reconstructed slices.

Another thread performs an optimized reconstruction pipeline. The conventional reconstruction pipeline typically consists of pre-processing operations (*e.g.* dark-flat-field correction, taking negative logarithm, ring-removal, *etc*.) and filtered back-projection. Computational complexity of the pre-processing step is lower than the complexity of the filtered back-projection step. Therefore, in what follows, we will focus on the reconstruction step and show its optimization for real-time evaluation.

Suppose that we have a filled projection buffer with the size *K*, where projections *d*(θ_*i*_, *s*, *z*) are given for each angle θ_*i*_ with *i* = 0,…, *K* − 1, signed distance *s* with respect to the rotation axis, and vertical position *z*. Let 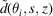 be a projection computed after applying all pre-processing procedures and filtering with the Shepp-Logan, Parzen, or other filter. The back-projection formula for recovering function *f*(*x*, *y*, *z*) applied after filtering can be written as follows, 

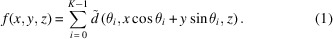

If we assume that the number of discrete points in each *x*, *y*, *z* direction is of the order of *N*, formula (1) can be directly evaluated with computational complexity 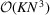 . There also exist methods with lower computational complexity [ 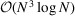 ], such as the Fourier-based or log-polar-based methods (Dutt & Rokhlin, 1993 ▸; Beylkin, 1998 ▸; Andersson *et al.*, 2016 ▸). These methods are significantly faster than the conventional back-projection in cases where the total number of angles for reconstruction is significantly bigger than the sample size in one dimension (*N*).

In this work, we use a similar approach as Buurlage *et al.* (2018 ▸) and directly evaluate formula (1) to obtain three arbitrary slice reconstructions for preset *x*, *y*, and *z*. We also added functionality for setting the rotation angle of each *x*, *y*, *z* slice to be able to follow different structures inside the sample. Implementation of this functionality is straightforward and only involves rotation of *x*, *y*, *z* planes by using a rotation matrix in 3D. The computational complexity for each of the three arbitrary reconstructions is 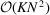 . The approach is more favorable compared with the aforementioned fast methods for two reasons. The first is that in many cases there is no need to reconstruct a full 3D data volume in real-time. Fast methods, such as the Fourier-based method, recover the object at each discrete point *x*, *y*, *z* by employing still significant computational resources and generating huge reconstruction volumes. For real-time streaming reconstruction a user can monitor only few 2D slices and make relatively fast decisions on when capturing data to disk or changing scanning parameters. In turn, 3D volumes are difficult to monitor without additional automatic data analysis.

Another reason for using the conventional reconstruction method is related to the optimal evaluation of (1) when projections are updated in streaming mode. Recall that reconstruction is performed for all projections in the buffer. Each upcoming projection and its unique ID are added to the buffer for further updates of the recovered volume. Let *f*_0_ be the recovered object from projections *d*_0_ representing a 180° interval. The new recovered volume *f*_1_ can be computed by using the whole 180° interval [see (1)], or, alternatively, by updating the part corresponding to the new projection set 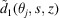 , *j* = 0,…, *K*′, 

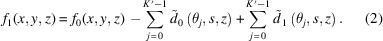

For 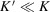 , evaluation by formula (2) is significantly faster than evaluation by (1). However, if acquisition is extremely fast and *K*′ > *K*/2, then formula (1) is computationally more favorable. In Section 3, we will provide computational time tables and analyze performance for different data acquisition scenarios.

The whole reconstruction pipeline is implemented by making use of the *CuPy* library (Okuta *et al.*, 2017 ▸) developed for GPU-accelerated computing with Python. The *CuPy* interface is highly compatible with *NumPy* and *SciPy*; in most cases it can be used as a drop-in replacement. In the proposed reconstruction implementation, all preprocessing procedures, such as dark-flat-field correction, taking negative logarithm, or filtering, were ported from a *NumPy* source code. In turn, back-projection, as the most computationally intensive part, was implemented via CUDA C kernels directly callable from the *CuPy* interface. Switching to GPU computations allowed us to achieve real-time reconstruction of three arbitrary slices through the sample. In some cases GPU memory is not enough to store the whole set of projections for a 180° interval. To address this issue we split projections into several datasets corresponding to different angles and process these datasets independently one by one. With this straightforward data splitting, one can use several GPUs for reconstruction and obtain almost linear performance gains with increasing number of GPUs.

**Figure 1:**
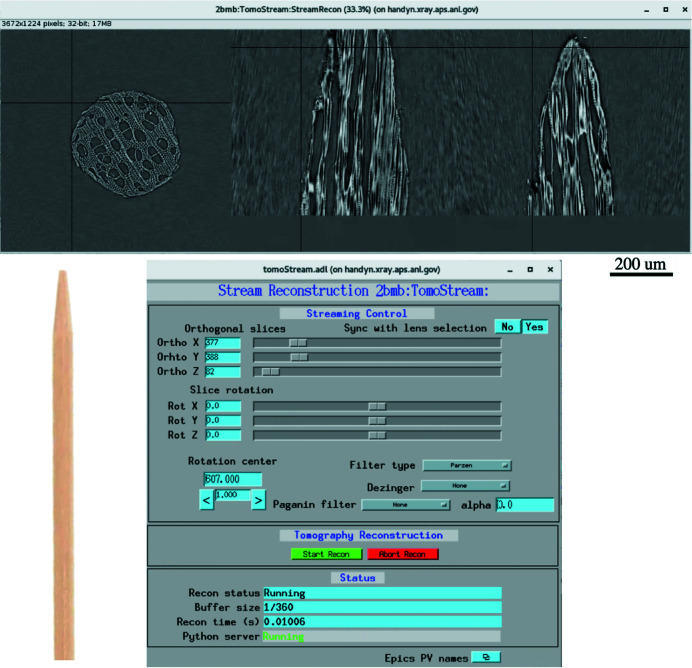
Real-time streaming reconstruction. Top: reconstructed orthogonal slices through a wood stick sample (concatenated as *z*, *y*, *x*). Bottom: MEDM screen for controlling reconstruction parameters. Solid black lines in the slices visualization indicate positions of orthogonal slices given in the MEDM screen. These slices can be arbitrarily tilted.

For real-time reconstruction visualization, the three arbitrary slices are concatenated into one 2D array and broadcast over the network as a structured pvAccess variable constructed with the *PvaPy* interface (Veseli, 2015 ▸). Visualization can be done at any computer in the network by making use of the EPICS NTNDA Viewer (Rivers, 2022*b* ▸) plugin for *ImageJ* (Schneider *et al.*, 2012 ▸). An example of three concatenated slices is shown in Fig. 1 ▸ (top). For the convenience of selecting the region of interest inside *ImageJ*, the slices are ordered as *z*, *y*, *x*. Solid black lines indicate the positions of each of the three arbitrary slices. The positions are given in the graphical user interface shown in the bottom panel of the same figure. The interface allows users to control reconstruction parameters such as the rotation axis location, FBP filter, *x*, *y*, *z* ortho-slice numbers, and their rotation angles. The user interface also allows to perform reconstruction with the phase retrieval procedure where the required parameters, such as the energy, pixel size, detector–sample distance, are automatically retrieved from EPICS PV variables associated with the scan, whereas the parameter α denoting the ratio between the phase δ and absorption β of the sample [see Paganin *et al.* (2002 ▸) for details] is adjusted manually. It is implemented as an EPICS MEDM (MEDM, 2022 ▸) screen where all parameters are associated with EPICS PV variables broadcasted over the network. It turns out that both three arbitrary slice visualization and control of parameters can be done remotely, with a regular computer or laptop.

We implemented the real-time streaming reconstruction and visualization of three arbitrary slices described above in a dedicated EPICS IOC called *tomoStream* (Nikitin & De Carlo, 2022 ▸). Demonstration Video 1 in the supporting information shows a test tomographic experiment with real-time streaming reconstruction and on-demand data capturing.

### 2.4. 3D zooming to regions of interest inside the sample, while in streaming mode

**Figure 2:**
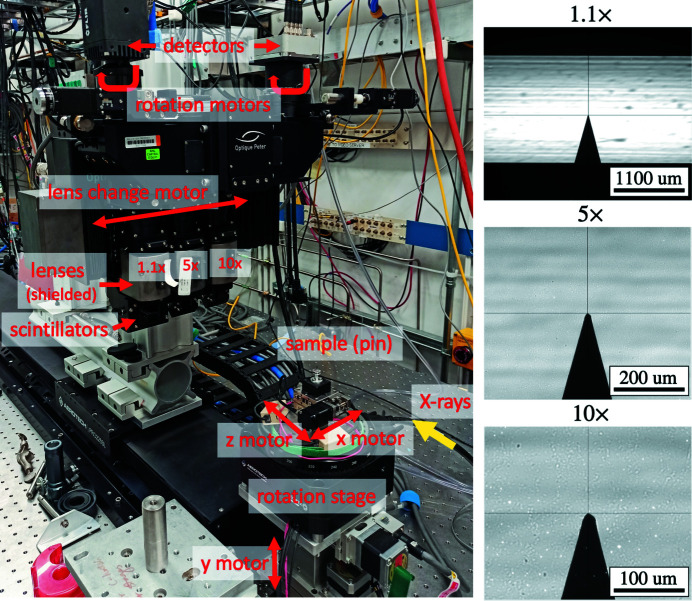
The Optique Peter microscope system at beamline 2-BM of the Advanced Photon Source (left). Result of automatic sample alignment for different magnification with *x*,*z* motors on the rotation stage, vertical *y* motor under the stage, and detector rotation motors on the top of the Optique Peter system (right).

At beamline 2-BM of the Advanced Photon Source we integrated the streaming system with an Optique Peter microscope to implement real-time zooming to regions of interest. The Optique Peter microscope system, see Fig. 2 ▸, consists of an automatic lens changer. The system has three lenses and three scintillators associated with each lens. The scintillator thickness influences photon flux and resolution limit in acquired projections. Therefore, a thinner scintillator is typically paired with a higher magnification lens, whereas a thicker scintillator is paired with a lower magnification lens.

In the current setup we operate with the following pairs: 1.1× magnification – 100 µm scintillator; 5× magnification – 50 µm scintillator; 10× magnification – 25 µm scintillator. All lenses are Mitutoyo Infinity Corrected Long Working Distance Objectives, scintillator type Crytur LuAG:Ce. Each scintillator converts the X-ray beam to visible light. Visible light is then redirected to the detector through a magnification lens and an array of mirrors. By using an additional mirror it is also possible to redirect the beam to the second detector having different acquisition frame rate and quantum efficiency. To demonstrate the zooming functionality in this work we operated with only one camera: Oryx 5.0 MP Mono 10GigE, 2448 × 2048 chip size, 3.45 µm pixel size by Teledyne FLIR LLC. A list of all cameras available in the Imaging Group of the APS is reported in Table 3.

The Optique Peter system does not guarantee that the sample image for different magnifications is aligned and located at the same position on the detector. Even after adjusting the lens change motor positions in a way that the rotation center is located in the middle of the detector field of view for each magnification, we still observe a misalignment caused by the different tilt angles of the mirrors redirecting visible light from scintillators to objectives. We also observed that the lens change operation causes a rotation of the image by a considerable angle. Instead of performing a manual calibration of the tilt angles for mirrors, we compensated the rotation axis misalignment by adjusting the sample stack motors and the image rotation by adjusting the camera rotation. All these misalignment offsets are repeatable and can be corrected by automatically adjusting the corresponding motors at each lens change by moving motors *x* (orthogonal to the beam), *z* (along the beam) mounted on the top of the rotation stage, *y* (vertical) located under the rotary stage, and the detector rotation, see Fig. 2 ▸ (left). In Fig. 2 ▸ (right) one can see test images of a pin for different magnifications where the system misalignment is compensated by the coordinated motion described above.

**Figure 3:**
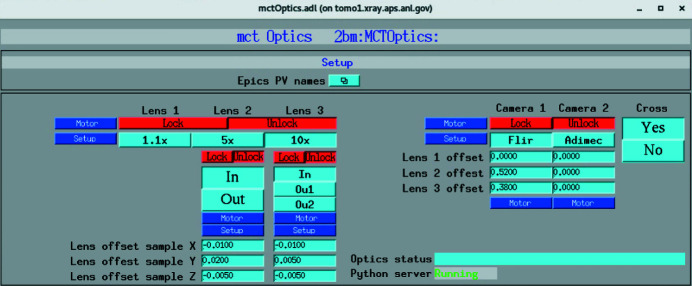
Control of the Optique Peter microscope system with three lens and two detector change. Once the sample *x*,*y*,*z* and detector rotation offsets are measured they are entered in the mctOptics setup screen as Lens offset sample *X*,*Y*,*Z* and Lens 1,2,3 offset. The instrument operator can change lens by pressing the lens selector button 1.1×, 5×, 10× while keeping the rotation axis aligned and locked at the same 3D sample location.

To hide the complexity of such coordinated motion during each lens change we developed a dedicated mctOptics (De Carlo, 2022*c* ▸) EPICS IOC, see Fig. 3 ▸.

It is not difficult to see that zooming to the region of interest can also be done by moving the sample on the rotation stage and vertically using the *x*,*z*,*y* motors. As an example, consider zooming to the region of interest centered at (500, 1224, 1500) in a (2448, 2448, 2048) volume reconstructed from tomographic data acquired with 1.1× magnification. To find the new motor positions, we simply subtract half of the detector size in each dimension from the ROI center, and multiply the result by the pixel size for 1.1× magnification. It follows that the *x*,*z*,*y* motors should be moved by (500 − 1224)/3.45 × 1.1 = −230.8 µm, (1224 − 1224)/3.45 × 1.1 = 0 µm, and (1500 − 1024)/3.45 × 1.1 = 151.8 µm, respectively. After changing the lens and correcting for the additional misalignment mentioned above, the center of the ROI will be located in the middle of the detector image. The same procedure is applied when zooming is done between other magnifications.

3D zooming to regions of interest has been efficiently combined with real-time streaming reconstruction of three arbitrary slices. In Fig. 1 ▸ (top), the solid black lines in the streaming reconstruction images indicate the position of ortho-slices selected with the Ortho (*X*,*Y*,*Z*) sliders of the streaming control MEDM screen. The tilt angle for each of the three orthogonal slices can also be adjusted in real-time. In our instrument, we assigned the location where the lines intersect each other as the region of interest which is kept in the same sample location during the lens change. This provides a simple and easy-to-use zoom-in/zoom-out capability available in streaming mode. To enable this feature, in the MEDM screen shown in Fig. 1 ▸ (bottom), we implemented a ‘Sync with lens selection’ button. When the button is set to ‘Yes’, we have synchronization between the slider selected ortho-slices and the region of interest for zooming. The sample is shifted at each magnification change so that the position of initial intersections for the solid black lines appears in the middle of all ortho-slices for each magnification level. During real-time streaming reconstruction, it is required to update the flat field after the 3D zooming is complete. To avoid re-taking flat fields each time, we decided to store flat fields for each magnification and read them from PV variables corresponding to different lenses. Users can update flat fields only when it is required by other experimental condition changes, *e.g.* when the exposure time is changed or the flat field drifted significantly.

Demonstration Video 2 in the supporting information shows the 3D zooming functionality during real-time streaming reconstruction.

## 3. Performance analysis

In this section we analyze the performance for each component of the proposed streaming tomography model and find out bottlenecks for the highest speed data processing.

To measure the performance of real-time tomographic reconstruction, we generate a set of projections and broadcast them to the reconstruction engine at different data rates. For this test, both the projection data generator and reconstruction engine are executed on the same machine equipped with a GPU. Projection size is 16-bit 1024 × 1024. In Table 1 ▸ we present results for two different GPUs, a very powerful and expensive NVidia Tesla A100 connected via PCI express v4.0, and a less powerful and relatively inexpensive NVidia Quadro RTX 4000 connected via PCI express 3.0. The number of projections in the GPU buffer used for reconstruction corresponds to the number of projection angles in a 180° interval and equal to 1024 for the Tesla A100, and 512 for the Quadro RTX 4000. The table shows that the total reconstruction time is always less than 1 s. However, in cases of very fast data acquisition some projections are missed and not used for reconstruction, which is indicated by the last column showing how many projections are stored in the buffer while one reconstruction is finished. For instance, if the projection rate is ≥5 GB s^−1^ for the machine with the Tesla A100 then the buffer is filled completely and new coming projections are replacing the elements in the buffer. However, if the data rate is ≤4 GB s^−1^ then the buffer is not filled during one reconstruction and all projections are used for processing. Results for low data rates, such as 2 and 3 GB s^−1^ for Tesla A100, demonstrate additional performance gain because computations are done using the optimized formula (2) from Section 2.3. The table shows that 4 GB s^−1^ and 1 GB s^−1^ are maximum projection data rates that machines with Tesla A100 and Quadro RTX 4000, respectively, can accommodate without missing any projection; nevertheless, it should be noted that in typical real experiments missing projections for real-time reconstruction in some cases may not lead to relevant losses of real-time information. Specifically, if a projection is missed, the reconstruction engine will use the projection corresponding to the same angle from the previous rotation. If the number of missed projections is not very high then the contribution by the incorrect projection is negligible and typically not seen by the eye. At the same time, since data capturing to HDF5 file is independent of projection broadcast and real-time reconstruction, see Section 2.1, the projections missed during streaming are fully captured by the on-demand data capturing pipeline and safely stored in an HDF5 file to be analyzed offline.

Another observation in Table 1 ▸ is the time distribution among different GPU data processing tasks when reconstruction is performed for the whole buffer. We observe that most of the time is spent on the FBP filter function that indeed becomes the most computationally expensive since it involves computing FFTs with a total computational complexity of 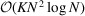 , where *K* is the number of angles, and *N* is the projection size in one dimension. CPU-GPU data copy is also computationally demanding and can be optimized by a faster data transfer link. One data transfer of a 1024 × 1024 × 1024 projection volume to Tesla A100 takes approximately the same time as the transfer of a 512 × 1024 × 1024 volume to Quadro RTX 4000, which is in agreement with the transfer speed difference between PCIe v4 and PCIe v3 links.

For the second performance test we analyzed data transfer speed over the network with the pvAccess channel. We created a pvAccess server on the detector machine and broadcast projections over the network at maximum speed. Any client connected via the pvAccess channel can read these projections and save them to a pre-allocated array. The pvAccess transfer speed is then measured as an average number of projections acquired in 1 s multiplied by the size of one projection in bytes. Table 2 ▸ shows the measured pvAccess transfer speed between several machines located at beamline 2-BM of the APS linked with different connection types. Machine ‘tomodata1’ acts as a server generating projections, *i.e.* a simulated detector. Other machines in the beamline network (‘tomo1’, ‘handyn’, and ‘tang’) act as clients reading projections from the network. We also measured the pvAccess channel and data reading overhead by setting ‘tomodata1’ as a client. The table shows the pvAccess transfer speed limit of 3.41 GB s^−1^ representing the overall protocol overhead. InfiniBand [Mellanox Technologies MT28908 Family (ConnectX-6) rate: 100 Gb s^−1^ (4X EDR)] connection between the detector and processing machine is favorable for the proposed streaming model and allows for keeping the speed at the level of 3.32 GB s^−1^. 10 Gb fiber and 1 Gb Ethernet connections confirm the declared speed of these connections.

In addition to performance tests, we list in Table 3 ▸ the specification of the fast detectors available at the Imaging Group of the APS. The table includes cameras with and without on-board memory. For the cameras without on-board memory we calculated maximum data rates according to the chip size, number of bits, and frames per second. Such cameras are connected to a detector computer with a link fast enough to handle the maximum frame rates. Cameras with on-board memory, in turn, are much faster and perform data copy to the detector machine whenever acquisition is carried out. In this case it is not possible to implement fast data broadcasting from the camera memory and thus provide a real-time reconstruction of projections coming at the maximum speed. However, here we consider streaming reconstruction with slower data acquisition speed to trigger the beginning of a fast dynamic process, followed by ultra-fast data capturing to the camera on-board memory without monitoring real-time sample states. For switching to fast data acquisition one can increase the rotation speed by following the procedure described in Section 2.2. By making use of the Optique Peter system, such as the one installed at beamline 2-BM (Fig. 2 ▸), it is also possible to automatically switch cameras between the two acquisition modes.

Finally, since for monitoring fast processes one needs to spin the sample at fast rotation speeds, we list in Table 4 ▸ the rotary stages available in the Imaging Group of the APS and allowing for 500 and 6000 rotations per minute, which is more than enough for capturing ultra-fast dynamic processes in 3D. All stages are controlled by the same Ensemble HLE10-40-A-MXH Aerotech Controller programmed using the Aerobasic language shown in Section 2.2.

## 4. Applications

**Figure 4:**
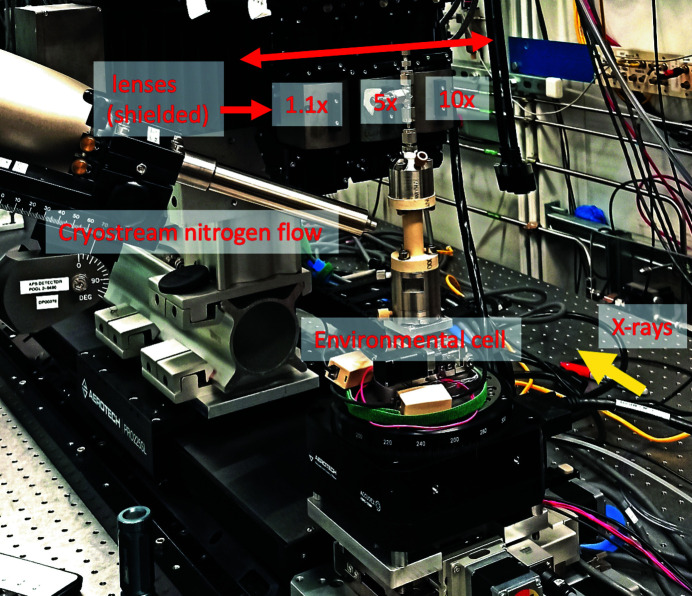
Setup for ice and gas hydrate formation experiments with real-time reconstruction and zooming to regions of interest.

In this section, we present applications of our streaming acquisition model with real-time reconstruction and 3D zooming to regions of interest. To demonstrate the efficacy of the proposed model we conducted synchrotron radiation tomography experiments on ice and methane gas hydrate formation in porous media (Nikitin *et al.*, 2020 ▸, 2021 ▸). The setup of the experiment at sector 2-BM of the Advanced Photon Source is presented in Fig. 4 ▸.

**Figure 5:**
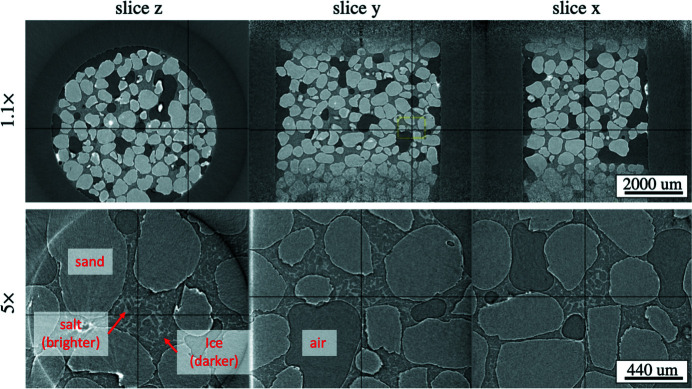
Real-time 3D zooming to the region of interest showing ice crystals formation surrounded by brighter brine. Rotation speed and exposure time are the same for both magnifications.

The formation process was carried out in an environmental cell (Fusseis *et al.*, 2014 ▸) packed with sand and 5% NaBr water solution. An Oxford 800 cryostream system was used for cooling the sample by a flow of nitrogen gas of low temperature. To monitor the process of ice formation we set a negative flow temperature (−40°C) in the cryostream system and performed scanning of the sample while it was continuously rotating. The top row of Fig. 5 ▸ shows real-time streaming reconstruction of orthogonal slices with 1.1× magnification. Note that the top and bottom part of the *x* and *y* slices contain noise because these regions are not illuminated by the beam due to its limited size in the vertical direction (similar beam deterioration can be observed in the pin image for 1.1× magnification in Fig. 2 ▸). In our future work, we plan to add a feature for manual cropping the camera field of view to decrease data sizes and optimize reconstruction. Solid black lines in the figure indicate positions of orthogonal slices selected by a user in the MEDM screen (Fig. 1 ▸). For convenience, we also agreed that in our implementation the center of the ROI for zooming corresponds to the intersection of these solid lines. After 5 min of cooling we observed regions where ice started to appear, forming brighter surrounding regions of brine with higher salt concentration (ice formation consumes only pure water leaving salt in the brine). We selected one of these regions and automatically zoomed into it with higher magnification. Reconstruction of orthogonal slices through the region of interest with 5× magnification is shown in the bottom row of Fig. 5 ▸. Here one can observe small salt structures between ice particles, whereas with 1.1× magnification these structures are non-distinguishable. Such imaging will be of great help in interpreting complicated dependence of acoustic properties (*i.e.* velocity and attenuation of seismic waves) of saline permafrost samples on temperature and pore-brine salinity observed in laboratory experiments, see Dou *et al.* (2016 ▸). Understanding these processes is important for developing seismic methods of mapping and monitoring saline permafrost for geotechnical applications.

With the on-demand data capturing option described is Section 2.1 we can save projections for a 180° interval representing this ROI to an HDF5 file, reconstruct and analyze it in more detail after streaming is done. So we can conveniently switch between different ROIs following our research needs. While streaming reconstruction is on-going we can easily select another ROI and capture data there. The proposed approach is clearly more effective than the conventional scanning where the ROI is blindly selected. In these scans we kept the same exposure time of 0.04 s, and angular step 0.5° for both magnifications. It should be noted that color bars in the two images of Fig. 5 ▸ are different. This is caused by the fact that projection data for 5× magnification also includes information about other parts of the sample, decreasing the contrast levels between different materials in reconstructions and amplifying phase artifacts on the material borders. The effect can be partially compensated by phase retrieval filtering (Paganin *et al.*, 2002 ▸). A better approach to address this issue will be discussed in Section 5.

Demonstration Videos 3 and 4 in the supporting information show real-time visualization of ice formation/melting in low and high resolution.

The ice formation experiment was a preliminary functionality test before a longer experiment for the methane gas hydrate formation in sand. The formation was done in the same environmental cell packed with sand and 5% NaBr water solution (brine concentration is 10% mass fraction in relation to sand). To simulate the gas hydrate growth we followed certain temperature and gas pressure conditions, see Sun *et al.* (2014 ▸). Specifically, we adjusted the cryostream cooling system to hold the temperature inside the environmental cell at the level of 7°C, and used a Teledyne ISCO D-Series syringe pump system to pressurize the cell with methane gas served via high-pressure tubes. The formation process took about 20 h. During this time we performed continuous vertical scanning of the environmental cell with 1.1× lens and periodically used 5× and 10× lenses to zoom into the regions of interest containing gas hydrate structures.

**Figure 6:**
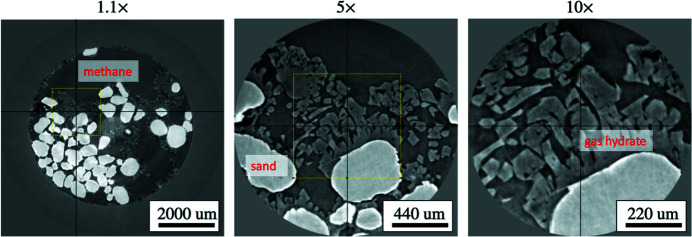
Reconstructed *z*-slices taken after real-time 3D zooming to the region of interest with formed gas hydrates. Rotation speed and exposure time were adjusted for each magnification change. The reconstruction procedure also involved phase-retrieval and circular mask filtering.

In Fig. 6 ▸ we demonstrate the results of real-time 3D zooming to image gas hydrate structures formed on sand particles at different resolution (using 1.1×, 5×, and 10× lenses). The resolution level for high magnifications allows for a more detailed analysis of the sand–hydrate interface and porosity inside gas hydrate volumes. After zooming we clearly see micro-porosity of the gas hydrate crystals. This will have a serious effect on the permeability of the hydrate-bearing porous sample at the macro-scale. In turn, changing perm­eability in hydrate-bearing sediment critically governs the growth and distribution of hydrates during their formation and gas production during the hydrate dissociation (Zhang *et al.*, 2020 ▸). Thus a pore-scale investigation of permeability changes during the hydrate formation/dissociation is important for planning gas production from hydrate reservoirs.

On the imaging side, note that such gas hydrate structures (as in Fig. 6 ▸) appear only in particular regions inside the environmental cell. Moreover, temperature and pressure variations may result in gas hydrate dissociation and structure modifications. Therefore, real-time zooming to regions of interest is highly beneficial compared with regular scanning of the whole sample, followed by offline reconstruction and manual selecting ROI for zooming inside a huge reconstructed volume.

Compared with the ice formation experiment where for scanning we used the same exposure time and angular step for both low and high magnifications, data acquisition parameters for the hydrate formation experiment were adjusted with respect to selected magnification. Exposure time and angular step pairs for different lenses were chosen as follows: 1.1°: 0.05 s, 0.25°; 5°: 0.2 s, 0.125°; 10°: 0.4 s, 0.125°. To enhance data quality the streaming reconstruction pipeline also contained phase retrieval (Paganin *et al.*, 2002 ▸) and circular mask filtering of the result.

## 5. Conclusions and outlook

Zooming to arbitrary regions of interest while performing streaming data collection and real-time reconstruction has been demonstrated using the three motorized lens system implemented by the Optique Peter microscope which is currently available at beamline 2-BM of the Advanced Photon Source. The current model allows for exposure time, rotation speed, and angular step size to be changed at any time during the data streaming process. These new features open a completely new way to optimize data collection parameters. Instead of ‘blind’ data capturing, one can select a relevant sample region directly looking at the streaming reconstruction, zoom into it, change exposure time and angular step to optimize reconstruction quality and, if the sample under study requires a faster camera, even swap detector in a few seconds, and finally, once all is set, capture data to an HDF5 file on-demand while streaming is on-going. By implementing a circular buffer storing images before the data saving trigger, we are always certain to capture the event of interest.

**Figure 7:**
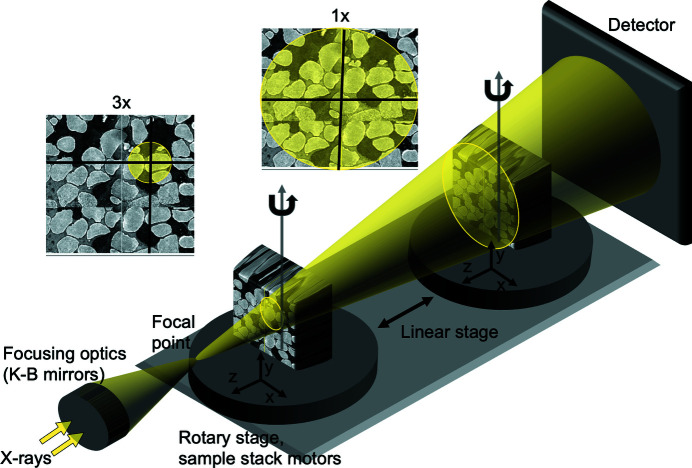
In an X-ray projection microscope the continuous zooming functionality is implemented by moving the sample along the cone beam: magnification is higher if the sample is closer to the focal point, and lower if the sample is closer to the detector.

As part of the APS Upgrade, the Imaging group will install an X-ray projection microscope, Fig. 7 ▸, where a zooming functionality is implemented by moving the sample along the cone beam. In this figure we schematically demonstrate 1× magnification by placing the sample stack closer to the detector, and 3× magnification by placing the stack closer to the beam focal point. The proposed streaming model can be directly implemented in the projection microscope to deliver continuous zooming reconstruction.

We conducted a series of performance tests for all components of the proposed streaming model, including real-time reconstruction and data transfer performance for different detector speeds. With the current software and hardware solution, it is possible to process data coming from the detector at 3–4 GB s^−1^, which corresponds to 1500–2000 projections of 16-bit 1024 × 1024 in size per second. Currently available detectors at beamline 2-BM broadcast data at a significantly lower speed (0.7–2 GB s^−1^). Detectors with on-board memory have higher speed; however, fast real-time data broadcasting is not possible. In the proposed model, slower detectors can still be used in combination with fast detectors, for instance to trigger ultra-fast data capturing.

The real-time X-ray tomographic microscopy we propose opens new possibilities for *in situ* characterization of micro-structure evolution in matter. As an application of the proposed technique, we considered a geological experiment for *in situ* ice and gas hydrate formation, where new insights about the formation process have been gathered. The technique could have a wide range of applications not only in geosciences but also in materials science, environmental science, and medical research. The method we developed will allow non-invasive imaging of objects to reveal their physical and chemical properties under different conditions and relate these properties to their density distribution in three-dimensional space at the micro- and nano-scales. Such relationships are key to understanding the properties of materials and could be used to identify minerals and oil-bearing rocks, look at *in situ* chemical reactions, distinguish between healthy and diseased tissue, or probe stress–strain gradients in manufactured components.

Despite the fact that the streaming tomographic model with real-time reconstruction already demonstrates several advantages compared with the conventional tomographic model, there is still a series of enhancements that may help in conducting more complex dynamic experiments. For example, the proposed tomographic scanning method requires the sample to continuously rotate, which may not be possible to implement when wires and water/gas lines are connected to the sample environment cell. In some cases this issue is solved using slip rings; however, this is not always possible. For these situations, we plan to implement streaming with a ‘back and forth’ scanning where an alternating sequence of 0–180° is followed by 180–0° rotation. In this case we expect to have a small data collection dead-time between each two 180° scans, where the rotary stage performs deceleration, change of direction, and acceleration. During these time periods, the reconstruction engine will pause and wait for the next set of projections. We also find it potentially possible to implement the streaming interlaced angular scanning protocol, in which a full tomographic scan is acquired with multiple sample rotations where projection angles are mod 2π different and uniformly cover the interval [0, 2π) (Mohan *et al.*, 2015 ▸; Zang *et al.*, 2018 ▸). With this protocol, a significant time gap between acquisition of two nearby angles, that should give similar data in a static case, allows to estimate corresponding shifts by using rigid image registration methods like cross-correlation. The cross-correlation procedure can also be evaluated in real-time. Another natural enhancement regards the local tomography problem (Kuchment *et al.*, 1995 ▸). When the samples are significantly larger than the detector field of view, *e.g.* in the 10× zoom-in magnification case, the projection data also include information from the portion of the sample that is outside of the field of view. With the automatic lens changing mechanism we can easily pad high resolution with low resolution projections and correct the high-resolution scans from local tomography artifacts (Xiao *et al.*, 2007 ▸). Finally, we plan to add AI-based methods (Schoonhoven *et al.*, 2020 ▸; Tekawade *et al.*, 2021 ▸) for detecting events and trigger data saving automatically. In fast-evolving dynamic systems, automatic segmentation, classification, and detection may allow for steering tomographic experiments, *e.g.* changing environmental conditions (pressure, temperature, charge) based on real-time sample states. AI-based steering techniques will play a very important role in future complex dynamic experiments at brilliant light sources.

## Supplementary Material

Demonstration of real-time streaming reconstruction and on-demand data capturing functionality. DOI: 10.1107/S1600577522003095/mo5255sup1.mp4

Demonstration of 3D zooming functionality with the Optique Peter system. DOI: 10.1107/S1600577522003095/mo5255sup2.mp4

Real-time visualization of ice formation in low resolution. DOI: 10.1107/S1600577522003095/mo5255sup3.mp4

Real-time visualization of ice melting in high resolution (zooming after low resolution reconstruction). DOI: 10.1107/S1600577522003095/mo5255sup4.mp4

## Figures and Tables

**Table:** Streaming projections acquired during one reconstruction are stored in a buffer of size 1024 for Tesla A100 and 512 for Quadro RTX 4000, representing an angular interval of 180°; if reconstruction is not fast enough then projections in the buffer are replaced by new ones. Lower table rows show GPU computational times for each reconstruction task.

NVidia Tesla A100, PCIe 4.0, CUDA v11.5		
Projection data rate	Total time for one reconstruction	Projections stored during one reconstruction
6 GB s^−1^	0.327 s	1024
5 GB s^−1^	0.321 s	1024
4 GB s^−1^	0.293 s	875
3 GB s^−1^	0.062 s	120
2 GB s^−1^	0.005 s	3

**Table:** 

CPU-GPU copy	Dark-flat-field correction	Negative logarithm	FBP filter	Back-projection
0.10 s	0.03 s	0.04 s	0.10 s	0.01 s

**Table:** 

NVidia Quadro RTX 4000, PCIe 3.0, CUDA v11.5		
Projection data rate	Total time for one reconstruction	Projections stored during one reconstruction
2.5 GB s^−1^	0.614 s	512
2 GB s^−1^	0.611 s	512
1.5 GB s^−1^	0.601 s	512
1 GB s^−1^	0.543 s	333
0.5 GB s^−1^	0.015 s	3

**Table:** 

CPU-GPU copy	Dark-flat-field correction	Negative logarithm	FBP filter	Back-projection
0.11 s	0.05 s	0.05 s	0.32 s	0.02 s

**Table 2:** Data transfer speed over the network using the pvAccess (PVA) channel for different connection types

Server machine name	Client machine name	Connection type	PVA transfer speed
tomodata1	tomodata1	Local	3.41 GB s^−1^
tomodata1	tomo1	InfiniBand†	3.32 GB s^−1^
tomodata1	handyn	10 Gb Fiber	1.08 GB s^−1^
tomodata1	tang	1 Gb Ethernet	0.11 GB s^−1^

† Mellanox Technologies MT28908 Family (ConnectX-6) rate: 100 Gb s^−1^ (4X EDR).

**Table 3:** Characteristics of fast detectors available at the Imaging Group of APS

Detector	Model	Chip size	Pixel size (µm)	Bits	Frames s^−1^	GB s^−1^
Adimec	Q-12A180	4000 × 3000	5.5	8–10	187	2.09
FLIR	ORX-10G-51S5M-C	2448 × 2048	3.45	8–12	167	0.78
PCO	Edge	2048 × 2048	6.5	16	100	0.78
FLIR	ORX-10G-310S9M	6464 × 4852	3.45	8–12	26	0.76
PCO	PCO DIMAX HS4	2000 × 2000	11	12	2277	†
Photron	Fastcam Nova S16	1024 × 1024	20	12	16000	†
Photron	Fastcam SA-Z	1024 × 1024	20	12	20000	†
Shimadzu	HPV-X2	400 × 250	32	10	10 × 10^6^	†

† These detectors use on-board camera memory to reach the specified maximum frames s^−1^. Data transfer to the controller computer varies depending on the camera-computer bus speed.

**Table 4:** Speed of rotary stage available at the Imaging Group of APS

Name	Manufacturer	Model	Speed (rev min^−1^)
Rotary stage	Aerotech	ABRS-150MP-M-AS	500
Rotary stage	Aerotech	ABS-250MP-M-AS	500
Rotary spindle	Aerotech	ABS2000-1000AS-RU	6000
